# On the energy of nonlinear water waves

**DOI:** 10.1098/rspa.2021.0544

**Published:** 2021-11

**Authors:** David Henry

**Affiliations:** School of Mathematical Sciences, University College Cork, Cork, Ireland

**Keywords:** nonlinear water waves, wave energy, exact solutions

## Abstract

This article presents results concerning the excess kinetic and potential energies for exact nonlinear water waves. In particular, it is proven, for periodic travelling irrotational water waves, that the excess kinetic energy density is always negative, whereas the excess potential energy density is always positive, in the steady reference frame. A characterization of the total excess energy density as a weighted mean of the kinetic energy along the wave surface profile is also presented.

## 1. Introduction

This article establishes results concerning the energy generated by nonlinear water waves. The analysis of water waves is an intriguing and challenging subject spanning a number of scientific disciplines—mathematical, physical, engineering [1–4]—and its intrinsic complexity is demonstrated by the range of fundamental theoretical questions that remain open despite centuries of intensive research [1,5]. Even in the setting of a perfect fluid (incompressible and inviscid), the governing equations are highly intractable, primarily due to strong nonlinearities, compounded by the presence of an unknown free-boundary. One classical approach that renders the governing equations more tractable, particularly with regard to applications (cf. [3]), involves linearizing the governing equations. However, while offering a useful first approximation to the water wave problem, this approach is only justified in the context of small amplitude water waves: for even moderate amplitude water waves, much of the underlying mathematical and physical structure is lost. Nonlinear waves possess a plethora of fundamental behaviours and physical characteristics that cannot be captured by, or divined from, linear approximations (cf. [1]).

Wave energy is a subject of great practical importance which is currently the focus of intense multidisciplinary research, particularly in relation to marine renewable energy [6]. The theory underlying ocean wave energy is a nascent field of scientific research, having been developed in recent decades, and suffers from the fundamental limitation that most of the state-of-the-art is strongly contingent on invoking linear approximations [6]. While there do exist some results characterizing energy properties for nonlinear water waves (see, for example, the ‘classical’ papers [7–11], and more recent developments [12,13]), due to the technical complexities inherent in nonlinear analysis, the literature is sparse in terms of both quantity and scope. Recent advances in mathematical analysis have enabled progress in tackling fundamental questions concerning nonlinear waves, and this article presents new results concerning the excess kinetic and potential energies for exact nonlinear periodic and travelling irrotational water waves.

For water waves travelling with uniform wavespeed, we can transform to a moving reference frame in which the resulting flow is steady. We define $E_{p}$ to be the excess mean potential energy of the wave in the moving frame over the value for the undisturbed flow (cf. (4.1)), and define $E_{k}$ to be the excess mean kinetic energy of the wave in the moving frame over the value of the undisturbed uniform flow with velocity components (c,0) (cf. (4.2)), where c is the uniform wavespeed. The mean is taken over a wave period, and hence, $E_{p}$ and $E_{k}$ are energy densities per unit length. For waves with relatively small amplitude (a/d≪1, where a is the amplitude and d is the mean water depth), explicit linear wave solutions exist, and we can directly compute the linear excess mean energies as follows:

1.1 $$E_{p}^{\text{lin}}=\frac{ga^{2}}{4}+O(a^{3})\;\text{and}\;E_{k}^{\text{lin}}=-\frac{a^{2}g}{4}+O(a^{3}),$$

where $O(a^{3})$ denotes terms of order $a^{3}$, and higher, in the asymptotic expansions, and g is the standard gravitational constant of acceleration (cf. appendix A). Hence, in the linear setting, for sufficiently small wave amplitudes, we have

1.2 $$E_{p}^{\text{lin}}>0\;\text{and}\;E_{k}^{\text{lin}}<0.$$

Furthermore, $E_{p}^{\text{lin}}$ and $E_{k}^{\text{lin}}$ have the same magnitudes (up to order $O(a^{2})$) and

1.3 $$E_{\text{tot}}^{\text{lin}}:=E_{p}^{\text{lin}}+E_{k}^{\text{lin}}=0,$$

where $E_{\text{tot}}^{\text{lin}}$ denotes the total linear excess energy at this level of linear approximation. The fact that relations (1.1)–(1.3) hold within the confines of linear water wave theory has important implications for practical applications [2–4], since they provide a convenient means of estimating the total wave energy. The measurement of kinetic energy is extremely difficult, however estimating the wave amplitude is much more feasible. Indeed, some recent theoretical work has established surface-profile recovery formulae for a range of nonlinear periodic water waves using measurements from submerged pressure transducers [14–17]. The formulae in (1.1) ensures that this procedure will yield an accurate estimate for the total wave energy when the wave amplitude is small.

In this article, we use an interplay between harmonic function theory and conformal mappings to establish the validity of relations (1.2) for exact periodic irrotational travelling wave solutions to the nonlinear governing equations for water waves. As a by-product, we derive a succinct formulation for the total excess energy of a nonlinear water wave, which can be expressed in terms of the mean kinetic energy along the wave surface profile, weighted by the wave surface profile itself.

## 2. Preliminaries

We consider the motion generated by two-dimensional steady periodic travelling waves propagating on the surface of an incompressible fluid under the restoring force of gravity. The occurrence of such waves can be observed in many different physical scenarios, for instance, in the regular undulation of the ocean surface known as ocean swell. It is well known that swell is hardly affected by viscosity [1,2,4], and so we focus on an inviscid flow with constant density ρ (for convenience, we set ρ=1). The system is described by Cartesian coordinates, for which (X,Y) denotes the horizontal and vertical coordinates and (u,v) gives the velocity field in these respective directions. To choose the reference frame, we let Y=−d (for some constant d>0) denote the location of the impermeable flat bed, while Y=η(X,t) represents the unknown free-surface, where η is even and periodic with respect to the spatial variable. The condition

2.1 $$\int_{0}^{\lambda }\eta (X,t)\;\text{d}X=0,$$

where λ>0 is the wavelength, fixes the mean water level at Y=0 and ensures that d is the fixed mean depth of the fluid. The amplitude a of the wave is the maximum deviation of the wave surface from the mean water level and may be defined at any fixed-time t by

2.2 $$a=\underset{X\in [0,\lambda ]}{\sup}\{\eta (X,t)\},$$

with this maximum value being attained at the wave crest.

**Remark 2.1.**

For nonlinear periodic waves observed in the sea, the amplitude a typically exceeds the distance between the wave trough and the mean water level. Accordingly, nonlinear waves tend to have sharper elevations and flatter depressions. The *wave height* is then defined to be the overall vertical change in height between the wave crest and the wave trough. In linear wave theory, wave surface profiles are sinusoidal (cf. the appendix) and the distances between the mean water level and the crest, respectively trough, coincide. The wave height is simply twice the wave amplitude in the linear setting.

The motion being steady implies an overarching functional dependence on the independent variables of the form (X−ct,Y), suggesting we transform to the reference frame moving with speed c via the change of variables

x=X−ct

and

y=Y.

The fluid domain in the moving frame, bounded above by the unknown free-surface y=η(x) and below by the flat bed y=−d, is denoted by

$$D_{\eta }=\{(x,y)\in R^{2}:-d<y<\eta (x)\},$$

and the governing equations for fluid motion in $D_{\eta }$ are given by the mass conservation equation

2.3*a* $$u_{x}+v_{y}=0\;\text{in}\;D_{\eta },$$

together with the Euler equation

2.3*b* $$\begin{array}{rl} (u-c)u_{x}+vu_{y} & =-P_{x}\\ \;\text{and}\;(u-c)v_{x}+vv_{y} & =-P_{y}-g\;\text{in}\;D_{\eta }, \end{array}\}$$

where P(x,y) is the pressure function and g is the standard gravitational constant of acceleration. At the free-surface, the kinematic and dynamic boundary conditions for the waves assume the form

2.3*c* $$v=(u-c)\eta_{x}\;\text{on }y=\eta (x)$$

and

2.3*d* $$P=P_{\text{atm}}\;\text{on }y=\eta (x),$$

where $P_{\text{atm}}$ is the (constant) atmospheric pressure: this dynamic boundary condition decouples the motion of the fluid from that of the air. At the rigid, impenetrable bed, the kinematic boundary condition is given by

2.3*e* v=0on y=−d.

In fluid mechanics, the local spin or rotation of an infinitesimal fluid element is measured by the vorticity, which is expressed by $\omega =u_{y}-v_{x}$ for two-dimensional motion. An inherent property of inviscid flows is that the vorticity of a given fluid element is preserved by the resultant fluid motion, and accordingly, a fluid mass which is initially irrotational will remain so for all further times. Bearing this in mind, it is physically quite reasonable to assume irrotationality in the fluid motion we analyse, a scenario which accords, for example, with regular ocean waves, or swell, entering a region of previously still water. This leads us to the additional irrotationality condition

2.3*f* $$u_{y}=v_{x}\;\text{throughout}\;D_{\eta }.$$

In the following, we analyse smooth exact solutions to the governing equations (2.3) for which η,u,v,P have period λ in the x−variable. Moreover, there is a single crest and trough per period, with $\eta^{\prime }(x)\neq 0$ except at the maximum (crest) or minimum (trough), and hence, the profile η is strictly decreasing from crest to trough. The functions η,u,P are symmetric, while v is antisymmetric about the crest. We choose the crest to lie on x=0, with the trough located at x=±λ/2. Such nonlinear waves are commonly referred to as Stokes' waves [18]. In addition, we assume that there is no constant underlying current, that is

2.4 $$\kappa =\int_{-\lambda /2}^{\lambda /2}u(x,-d)\;\text{d}x=0.$$

The flow being irrotational implies that the physical system is conservative, since relation (2.3*f*) enables the definition (up to a constant) of a velocity potential ϕ(x,y) by way of the relations

2.5 $$\phi_{x}:=u-c\;\text{and}\;\phi_{y}:=v.$$

It follows from (2.3*a*) and relation (2.5) that ϕ is a harmonic function. Fixing ϕ=0 on the crest line, we can express

$$\phi (x,y)=\int_{0}^{x}[u(l,-d)-c]\;\text{d}l+\int_{-d}^{y}v(x,s)\;\text{d}s,$$

from which we deduce that ϕ(x,y)+cx has period λ in x, ϕ is odd in the x−variable and vanishes at x=0, and ϕ(λn,y)=−cλn for any integer n. Relation (2.3*a*) enables the definition (up to a constant) of a stream function ψ by

2.6 $$\psi_{y}:=u-c=\phi_{x}\;\text{and}\;\psi_{x}:=-v=-\phi_{y}.$$

We fix the constant by setting ψ=0 on y=η(x). If we define

2.7 $$m=\int_{-d}^{\eta (x)}(u(x,y)-c)\;\text{d}y,$$

where m is the relative mass flux of the fluid motion, then it follows from direct calculation that m is an invariant of the flow. An important consequence of the irrotationality condition (2.3*f*) is that the stream function ψ and hence also $\psi_{y}$ and u are harmonic functions throughout the fluid domain $D_{\eta }$, and it can be shown by direct calculation that the level sets of ψ(x,y) are streamlines of the fluid motion. The strong maximum principle for harmonic functions [19] implies that m≠0, unless the flow is trivial. Since ψ=0 on y=η, applying the strong maximum principle for harmonic functions to ψ, and in turn $\psi_{y}$, we can infer that m<0, with

2.8 $$\psi_{y}=u(x,y)-c<0\;\text{in}\;\bar{D_{\eta }}.$$

Relation (2.8) expresses the absence of stagnation points throughout the fluid, which is a physically reasonable assumption for water waves without underlying currents containing strong non-uniformities and which are not near breaking. Indeed, in this setting, the maximal horizontal velocities u have a magnitude typically around 10% of the wavespeed. It can be shown that the maximum of the harmonic function u is attained precisely at the wave crest, cf. [1]. The limiting case whereby the magnitude of u approaches the wavespeed c at the crest, known as Stokes’ extreme wave, is a mathematically fascinating nonlinear wave that possesses a singular point at its angular wave crest, cf. [1,17]. Finally, (2.6) implies that ψ=−m on y=−d and expressing

$$\psi (x,y)=-m+\int_{-d}^{y}(u(x,s)-c)\;\text{d}s,$$

it is clear that ψ is also periodic with respect to the x−variable, with period λ. From (2.3*b*), we derive Bernoulli’s law, which states that the expression

2.9 $$E:=|\nabla \psi {|}^{2}+2gy+2P$$

is constant throughout the fluid. Hence, the governing equations (2.3) can be reformulated in terms of the stream function in the moving frame as an elliptic equation with nonlinear boundary conditions, given by the following free-boundary problem:

2.10*a* $$\begin{array}{rl} \Delta \psi & =0\;\text{in}\;D_{\eta }, \end{array}$$

2.10*b* $$\begin{array}{rl} |\nabla \psi {|}^{2}+2g(\eta +d) & =Q\;\text{on}\;y=\eta (x), \end{array}$$

2.10*c* $$\begin{array}{rl} \psi & =0\;\text{on}\;y=\eta (x) \end{array}$$

2.10*d* $$\begin{array}{rl} \psi & =-m\;\text{on}\;y=-d. \end{array}$$

The physical constant Q, referred to as the ‘relative hydraulic head’, satisfies Q>0.

## 3. Wavespeed determination

From the viewpoint of mathematical analysis, the governing equations formulated in (2.10) are advantageous primarily due to the steady fluid motion which prevails in the moving frame of reference. However, one practical issue which arises from working in the steady reference frame concerns the determination of the wavespeed c, which does not appear explicitly in system (2.10). The issue of determining the wavespeed from system (2.10) is a surprisingly complex matter both from the mathematical [1,20] and physical [21] perspectives. Indeed, there exists no canonical definition of the wavespeed, but rather there are two standard approaches to characterizing the wavespeed known as Stokes’ first, and second, definitions. From relations (2.4) and (2.6), the wavespeed c can be determined by the expression

3.1 $$c=-\frac{1}{\lambda }\int_{-\lambda /2}^{\lambda /2}\psi_{y}(x,y_{0})\;\text{d}x>0.$$

This corresponds to Stokes’ first definition, whereby the wavespeed is defined to be the mean horizontal velocity of the fluid in the moving frame of reference for which the wave is stationary. It is easily seen that expression (3.1) is independent of the (fixed) depth $y_{0}$ beneath the wave trough level. Stokes’ second definition sets the wavespeed $\tilde{c}$ equal to the depth-averaged horizontal fluid velocity in the moving frame, giving (by (2.6) and (2.7))

3.2 $$\tilde{c}=-\frac{1}{\lambda d}\int_{-\lambda /2}^{\lambda /2}\int_{-d}^{\eta (x)}\psi_{y}(x,y_{0})\;\text{d}y\;\text{d}x=-\frac{m}{d}>0.$$

These definitions agree for linear waves, in the sense that $c=c_{0}$, where $c_{0}$ is the wavespeed given by the linear dispersion relation (A 3), and $c=\tilde{c}+O(a^{2})$. However, the definitions (3.1) and (3.2) do not align in the nonlinear wave setting. In [20], it was shown that

3.3 $$c-\tilde{c}=\frac{1}{\lambda d}\int_{-\lambda /2}^{\lambda /2}\eta (x)(u(x,\eta (x))-c)\;\text{d}x=\frac{1}{\lambda d}\int_{-\lambda /2}^{\lambda /2}\eta (x)u(x,\eta (x))\;\text{d}x>0,$$

where the second equality follows from an implementation of (2.1). Hence, the mean horizontal wavespeed c, defined by (3.1), exceeds the mass-transport wavespeed $\tilde{c}$, defined by (3.2), in general. These relations for nonlinear waves play a fundamental role in the energy considerations below.

## 4. Wave energy

As a fluid moves, it must possess energy. For surface gravity waves on an inviscid fluid, the total energy consists of the potential energy (resulting from the displacement of the mass of water from a position of equilibrium under the gravitational field) and the kinetic energy (due to the motion of the water particles throughout the fluid), cf. [2,4]. Potential energy is the capacity for doing work due to the position of a body, while kinetic energy is the capacity for doing work by reason of the motion of a body. For ocean swell, the effects of viscosity are quite negligible [1,2,4], which accounts for the remarkable property of water waves to propagate over very long distances with relatively little loss of energy. Indeed, this property of persistence of energy is the primary motivation behind the desire to harness wave energy (cf. the discussions in [6]). A hallmark of inviscid fluids is the absence of dissipating effects, and accordingly, one expects a conservation of the total energy and a resulting balance between fluctuations in both the potential and kinetic energy [8–10]. In the setting of linear theory, the balance between both forms of energy is categorical, since the potential and kinetic energies can be explicitly computed (see the appendix), and an equipartition between mean potential and kinetic energies prevails. In the nonlinear regime, matters are, unsurprisingly, not as clear-cut, and indeed little is known for the fully nonlinear exact equations.

For fluids with infinite extent, it is meaningless to discuss the total energy possessed by the fluid, rather we must consider suitably defined local energy densities. For periodic surface gravity waves, we define the excess potential energy per unit horizontal area over the value for the flow with an undisturbed free-surface (η(x)≡0) by

4.1 $$E_{p}=\frac{1}{\lambda }\int_{0}^{\lambda }\int_{-d}^{\eta (x)}gy\;\text{d}y\;\text{d}x-\frac{1}{\lambda }\int_{0}^{\lambda }\int_{-d}^{0}gy\;\text{d}y\;\text{d}x,$$

whereas the excess kinetic energy per unit horizontal area over the value for the undisturbed uniform flow (u−c,v)=(−c,0) is given by

4.2 $$E_{k}=\frac{1}{2\lambda }\int_{0}^{\lambda }\int_{-d}^{\eta (x)}({\left(u-c\right)}^{2}+v^{2})\;\text{d}y\;\text{d}x-\frac{1}{2\lambda }\int_{0}^{\lambda }\int_{-d}^{0}c^{2}\;\text{d}y\;\text{d}x.$$

### (a) Excess potential energy

Our first result concerning the excess potential energy $E_{p}$ follows immediately from the definition (4.1) and reflects the fact that both a raised and depressed free-surface serve to increase the potential energy to an amount proportional to the square of the displacement from the mean water level. The raised surface increases the potential energy through adding new fluid above the position of the mean water level, whereas a depressed surface increases the potential energy through the removal of fluid beneath the mean water level.

**Proposition 4.1.**

*The excess potential energy per unit horizontal area*, $E_{p}$, *can be expressed as follows*:

4.3 $$E_{p}=\frac{g}{\lambda }\int_{0}^{\lambda /2}\eta^{2}(x)\;\text{d}x.$$

*Hence*, $E_{p}>0$ *for all* (*non-trivial*) *water wave solutions of the nonlinear governing equations* (*2.3*).

**Proof.**

It is extremely straightforward to show that the presence of free-surface waves increases the potential energy of a flow. Expression (4.1) reduces to

$$E_{p}=\frac{1}{\lambda }\int_{0}^{\lambda }\int_{0}^{\eta (x)}gy\;\text{d}y\;\text{d}x=\frac{g}{2\lambda }\int_{-\lambda /2}^{\lambda /2}\eta^{2}(x)\;\text{d}x>0,$$

with equality holding only in the absence of free-surface waves (η≡0), in which case the potential energy for the flow is minimized. Expression (4.3) follows from symmetry considerations.

To get an estimate for the excess potential energy for small amplitude waves, we adapt the linear Ansatz (A 2) to have $\eta (x)=a\cos(kx)+O(a^{2})$, where k=2π/λ denotes the wave number. The expression (4.3) can be directly computed to give

4.4 $$E_{p}^{\text{lin}}=\frac{ga^{2}}{4}+O(a^{3}).$$

### (b) Excess kinetic energy

Our next result concerning the excess kinetic energy is highly non-trivial and requires some subtle analysis. This result states that the presence of waves serves to decrease the excess kinetic energy in the moving frame.

**Proposition 4.2.**

*The excess kinetic energy per unit horizontal area*, $E_{k}$, *can be expressed as follows*:

4.5 $$E_{k}=-\frac{c}{\lambda }\int_{0}^{\lambda /2}\eta (x)u(x,\eta (x))\;\text{d}x.$$

*Hence*, $E_{k}<0$ *for all* (*non-trivial*) *water wave solutions of the nonlinear governing equations* (*2.3*).

The sign of this inequality concurs with that predicted by the linear approximation for small amplitude waves. In the linear regime (see appendix A), expression (4.2) becomes

4.6 $$E_{k}^{\text{lin}}=\frac{1}{2\lambda }\int_{0}^{\lambda }\int_{-d}^{0}(u^{2}+v^{2})\;\text{d}y\;\text{d}x-\frac{c}{\lambda }\int_{0}^{\lambda }\int_{-d}^{\eta (x)}u\;\text{d}y\;\text{d}x+\frac{1}{2\lambda }\int_{0}^{\lambda }\int_{0}^{\eta (x)}c^{2}\;\text{d}y\;\text{d}x+O(a^{3}).$$

The first term in (4.6) is given by (A 6), the third term is zero due to (2.1) and the second term in (4.6) is found (using (A 2)) to be

$$\begin{array}{rl} & \frac{c}{\lambda }a\omega \int_{0}^{\lambda }\int_{-d}^{\eta (x)}\frac{\cosh(k(d+y))}{\sinh(kd)}\cos kx\;\text{d}y\;\text{d}x=\frac{c\omega }{\lambda k}a\int_{0}^{\lambda }\frac{\sinh(k(d+\eta (x)))}{\sinh kd}\cos kx\;\text{d}x\\ & \;=\frac{c\omega }{\lambda }\frac{\cosh kd}{\sinh kd}a^{2}\int_{0}^{\lambda }\cos^{2}kx\;\text{d}x+O(a^{3})\overset{\left(\text{A}\;3\right)}{=}\frac{a^{2}g}{2}+O(a^{3}). \end{array}$$

Hence, (4.6) can be explicitly computed in the linear setting to get

4.7 $$E_{k}^{\text{lin}}=\frac{a^{2}g}{4}-\frac{a^{2}g}{2}+O(a^{3})=-\frac{a^{2}g}{4}+O(a^{3}).$$

While $E_{k}^{\text{lin}}$ given by (4.7) is clearly negative for linear water waves with sufficiently small amplitude a, there is no obvious reason to conclude that a similar inequality must hold for nonlinear waves. That is the aim of proposition 4.2, which we now prove.

**Proof.**

In the nonlinear wave regime, we work as follows. Define the hodograph change of variables

4.8 $$\begin{array}{rl} & q=-\phi (x,y)\\ \;\text{and}\; & p=-\psi (x,y), \end{array}\}$$

for ψ and ϕ the stream function and velocity potential. Transformation (4.8) is conformal, since ϕ and ψ are harmonic conjugates, and furthermore, it transforms the fluid domain with an unknown free-boundary into the fixed rectangular domain R=[0,cλ]×[−m,0]. Defining the height function by

h(q,p)=y+d,

we note that h is harmonic in terms of the (q,p) variables since (4.8) is a conformal mapping. We have

$$\partial_{q}=h_{p}\partial_{x}+h_{q}\partial_{y}$$

and

$$\partial_{p}=-h_{q}\partial_{x}+h_{p}\partial_{y},$$

with

$$\partial_{x}=(c-u)\partial_{q}+v\partial_{p}$$

and

$$\partial_{y}=-v\partial_{q}+(c-u)\partial_{p},$$

while

4.9 $$h_{q}=-\frac{v}{{\left(c-u\right)}^{2}+v^{2}}=-\frac{\partial x}{\partial p}=\frac{\partial y}{\partial q}\;\text{and}\;h_{p}=\frac{c-u}{{\left(c-u\right)}^{2}+v^{2}}=\frac{\partial x}{\partial q}=\frac{\partial y}{\partial p}.$$

From relations (4.9), we can re-express the excess kinetic energy (4.2) as follows:

4.10 $$\begin{array}{rl} E_{k} & =\frac{1}{2\lambda }\int_{0}^{\lambda }\int_{-d}^{\eta (x)}({\left(u-c\right)}^{2}+v^{2})\;\text{d}y\;\text{d}x-\frac{c^{2}d}{2}\\ & =\frac{1}{2\lambda }\int_{0}^{c\lambda }\int_{m}^{0}\frac{1}{h_{p}^{2}+h_{q}^{2}}\left|\frac{\partial (x,y)}{\partial (q,p)}\right|\;\text{d}p\;\text{d}q-\frac{c^{2}d}{2}\\ & =\frac{1}{2\lambda }\int_{0}^{c\lambda }\int_{m}^{0}\frac{1}{h_{p}^{2}+h_{q}^{2}}(h_{p}^{2}+h_{q}^{2})\;\text{d}p\;\text{d}q-\frac{c^{2}d}{2}=-\frac{c}{2}(m+cd)=-\frac{cd}{2}(c-\tilde{c}). \end{array}$$

The last equality in (4.10) follows from relation (3.2). Using relation (3.3) in (4.10), we conclude that

$$E_{k}=-\frac{c}{2\lambda }\int_{-\lambda /2}^{\lambda /2}\eta (x)u(x,\eta (x))\;\text{d}x<0.$$

Expression (4.5) follows from symmetry considerations.

We note that implementing the expressions for the linear wave solution (A 2), it can be seen that expression (4.5) matches (4.7) in the linear regime.

### (c) Total excess energy

Let us now define the total excess energy by $E_{\text{tot}}=E_{p}+E_{k}$. Following propositions 4.1 and 4.2, the total excess energy $E_{\text{tot}}$ can be characterized for nonlinear waves in terms of the following succinct expression.

**Proposition 4.3.**

*The total excess energy for nonlinear waves is given by*

4.11 $$E_{\mathrm{tot}}=-\frac{1}{2\lambda }\int_{0}^{\lambda /2}\eta (x)(u^{2}(x,\eta (x))+v^{2}(x,\eta (x)))\;\text{d}x.$$

**Proof.**

For nonlinear water waves, (4.3) and (4.5) give

$$\begin{array}{rl} E_{\text{tot}}=E_{p}+E_{k} & =\frac{1}{2\lambda }\int_{-\lambda /2}^{\lambda /2}\eta (x)(g\eta (x)-cu(x,\eta (x)))\;\text{d}x,\\ & =-\frac{1}{4\lambda }\int_{-\lambda /2}^{\lambda /2}\eta (x)(u^{2}(x,\eta (x))+v^{2}(x,\eta (x)))\;\text{d}x. \end{array}$$

The last equality follows from the Bernoulli relation (2.10*b*) combined with an application of (2.1). Expression (4.11) now follows from symmetry considerations.

The total excess energy for water waves can be expressed as the mean of the kinetic energy along the wave surface profile, weighted by the wave surface profile itself. While this expression pertains to the moving reference frame, interestingly it involves an evaluation of the kinetic energy for the velocity field of the fixed coordinate system. For linear water waves, expressions (4.4) and (4.7) show that excess potential and kinetic energies have the same magnitudes, but different signs, at the first order of approximation ($O(a^{2})$). Hence, the total excess energy is zero for linear water waves,

$$E_{\text{tot}}^{\text{lin}}=E_{p}^{\text{lin}}+E_{k}^{\text{lin}}=0.$$

This corresponds with expression (4.11), which is zero (at order $O(a^{2})$) when evaluated for the linear wave solutions (A 2).

For nonlinear waves, it is highly non-trivial to analytically ascertain the sign of this quite elegant relation. Determining the sign of (4.11) would provide insight into whether the kinetic or potential energies predominate for a given wave solution. Regarding the wave surface profile η(x), we know that η(0)>0, and η(λ/2)<0, with $\eta^{\prime }(x)<0$ for x∈(0,λ/2). As noted in remark 2.1, nonlinear waves tend to have sharper crest elevations and flatter depressions compared with linear waves, which has obvious implications for the weighting provided by the η(x) term in (4.11). Regarding the velocity field, some rigorous results do exist concerning monotonicity properties of the horizontal velocity component u along streamlines. In particular, it can be shown that $\partial_{x}u(x,\eta (x))<0$ for x∈(0,λ/2) (cf. [1,22–26]). However, very little is known analytically about the behaviour of the vertical velocity v, and rigorously establishing qualitative properties for v along the free-surface has heretofore proven elusive (cf. [27] for numerical investigations that provide some insight into this question). Rigorous results concerning monotonicity properties of the kinetic energy for nonlinear waves do exist [7,12,13]; however, these establish an exponential decrease of the kinetic energy with respect to vertical depth (for fluid motion beneath the wave trough) and do not pertain to the behaviour along the free-surface. Relation (4.11) is therefore a new expression that is worthy of further analytical, and numerical, investigations for nonlinear wave solutions.

## 5. Discussion

We have considered the excess potential ($E_{p}$), excess kinetic ($E_{k}$) and total excess ($E_{\text{tot}}$) energies for nonlinear periodic travelling waves. It is immediately apparent for non-trivial waves that the excess potential energy $E_{p}$ must be strictly positive. It is established in proposition 4.2 that the excess kinetic energy $E_{k}$ also obeys a strict sign, which is negative. While this result is known classically for linear wave solutions with sufficiently small amplitudes, it is certainly not self-evident that a similar result must apply universally for larger amplitude, and nonlinear, waves.

For linear waves, it can be demonstrated by explicit computations that there is an equipartition of energy, in the sense that the magnitudes of $E_{p}^{\text{lin}}$ and $E_{k}^{\text{lin}}$ are equal, and the total linear excess energy $E_{\text{tot}}^{\text{lin}}$ vanishes. While propositions 4.1 and 4.2 assert that $E_{p}$ and $E_{k}$ have strictly opposite signs for wave amplitudes beyond the linear regime, there are no comparable explicit expressions which relate their magnitudes. In proposition 4.3, we derive a new expression (4.11) for the total excess energy $E_{\text{tot}}$ of a nonlinear periodic travelling wave, whose value provides a measure of the balance between the excess potential and kinetic energies. An equipartition between the excess energies prevails only if (4.11) vanishes; if $E_{\text{tot}}$ is positive, then the excess potential energy $E_{p}$ predominates, and *vice versa*.

Together with (4.11), in the course of our analysis we have derived expressions for the excess potential energy, (4.3), and excess kinetic energy, (4.5), of which (4.5) is apparently new. These expressions require the evaluation of various integrals along the wave surface, and it is expected that they will be amenable to numerical evaluation. The numerical computation of these expressions for various wave solutions presents a direct opportunity to gauge how the various excess energy densities change as the nonlinearity of the wave solutions increase. It can also be hoped that these expressions may yield further insights by way of rigorous mathematical analysis in the future.

## Data accessibility

This article has no additional data.

## Appendix A

**(a) Linear gravity water waves**

The governing equations (2.3), expressed in terms of physical variables (X,Y), can be non-dimensionalized using the transformation

$$X\mapsto \lambda X,\;Y\mapsto \text{d}Y,\;t\mapsto \frac{\lambda }{\sqrt{gd}}t,\;u\mapsto u\sqrt{gd},\;v\mapsto v\frac{\text{d}\sqrt{gd}}{\lambda },\;\eta \mapsto a\eta ,$$

where λ is a typical wavelength and a is a typical amplitude of the wave. We avoid new notation by replacing, for example, X by λX, with X now being the non-dimensionalized variable. We set the constant water density ρ=1, and the pressure in the new non-dimensional variables is given by $P=P_{0}-gYd+gpd$, where the non-dimensional pressure variable p measures the deviation from the hydrostatic pressure distribution. Scaling the non-dimensional variables p↦ϵp, (u,v)↦ϵ(u,v), where ϵ=a/d is the amplitude parameter (and again avoiding the introduction of new variables) leads to the following boundary value problem in non-dimensional variables:

A 1 $$\begin{array}{rl} & u_{t}+\epsilon (uu_{X}+vu_{Y})=-p_{X},\\ & \delta^{2}\{v_{t}+\epsilon (uv_{X}+vv_{Y})\}=-p_{Y},\\ & u_{X}+v_{Y}=0,\\ & v=\eta_{t}+\epsilon u\eta_{X}\;\text{and}\;p=\eta \;\text{on}\;Y=\epsilon \eta \\ & v=0\;\text{on}\;Y=-1. \end{array}\}$$

Here, δ=d/λ is the shallowness parameter. The linearized problem is obtained by letting ϵ→0 in (A 1), which may be solved in terms of travelling wave solutions. Returning to the original physical variables, using the change of variables

$$X\mapsto \frac{X}{\lambda },\;Y\mapsto \frac{Y}{d},\;t\mapsto t\frac{\sqrt{gd}}{\lambda },\;u\mapsto \frac{u}{\sqrt{gd}},\;v\mapsto v\frac{\lambda }{\text{d}\sqrt{gd}},\;\eta \mapsto \frac{\eta }{a},$$

the linear wave solution in terms of the physical variables is given by

A 2 $$\begin{array}{rl} & \eta (t,X)=a\cos(kX-\omega t),\\ & u(t,X,Y)=a\omega \frac{\cosh(k(d+Y))}{\sinh(kd)}\cos(kX-\omega t),\\ & v(t,X,Y)=a\omega \frac{\sinh(k(d+Y))}{\sinh(kd)}\sin(kX-\omega t)\\ \;\text{and}\; & P(t,X,Y)=P_{0}-gY+ag\frac{\cosh(k(d+Y))}{\cosh(kd)}\cos(kX-\omega t), \end{array}\}$$

where k=2π/λ is the wavenumber, $\omega =\sqrt{gk\tanh\left(kd\right)}$ the frequency and the speed $c_{0}$ of the linear wave is determined by the dispersion relation

A 3 $$c_{0}=\frac{\omega }{k}=\sqrt{g\frac{\tanh(kd)}{k}}.$$

It can be shown that $c_{0}$ matches Stokes' first definition of the wavespeed (3.1): $c_{0}=c$. An important quantity in linear energy considerations is the group velocity, which is defined as follows:

A 4 $$c_{g}=\frac{\text{d}\omega }{dk}=\frac{g}{2\omega }\left[\tanh(kd)+\frac{kd}{\cosh^{2}(kd)}\right].$$

**(b) Wave energy: density and flux**

**(i) Potential energy V**

The potential energy V of a water wave at a fixed point X, measured relative to the undisturbed water level Y=0, is $V=\int_{0}^{\eta (X,t)}gY\;\text{d}Y=\frac{1}{2}g\eta^{2}$. For the linear wave solution given by (A 2), η(x,t)=acos⁡(kX−ωt), we get

$$V=\frac{1}{2}ga^{2}\cos^{2}(kX-\omega t),$$

and we see that the potential energy is a quantity which varies with respect to both x and t. The average of V over a wave period, denoted $\bar{V}$, will be independent of both space and time and so serves as a more useful measure of the potential energy of the wave. Using the fact that the average of $\cos^{2}(kX-\omega t)$ over a period is 1/2 (as is $\sin^{2}(kX-\omega t)$), we compute the potential energy density

A 5 $$\bar{V}=\frac{1}{4}ga^{2}.$$

**(ii) Kinetic energy T**

The kinetic energy T at a fixed point X is given (to $O(a^{2})$) by

$$T=\frac{1}{2}\int_{-d}^{0}(u^{2}+v^{2})\;\text{d}Y=\frac{a^{2}k^{2}g^{2}}{4\omega^{2}\cosh^{2}(kd)}\left[\frac{1}{2k}\sinh(2kd)+d\cos[2(kX-\omega t)]\right].$$

Taking the average of T over a wave period, $\bar{T}$ gives the kinetic energy density

A 6 $$\bar{T}=\frac{a^{2}k^{2}g^{2}}{8k\omega^{2}\cosh^{2}(kd)}\sinh(2kd)=\frac{a^{2}k^{2}g^{2}}{4k\omega^{2}}\tanh(kd)\overset{\left(\text{A}\;3\right)}{=}\frac{a^{2}g}{4}.$$

**(iii) Total energy E**

The total energy is given by E=V+T, with mean value

$$E=\bar{E}=\bar{V}+\bar{T}=\frac{1}{2}ga^{2}.$$

There is an equipartition of energy for a linear water wave between the (mean) potential and kinetic energy densities.

**(iv) Energy propagation**

The energy flux across a vertical plane, in the direction of motion of the wave crests (positive X−direction) is given by

$$\begin{array}{rl} E_{f} & =\text{Rate of doing work on the surface of the plane}+\text{convection of energy}\\ & =\int_{-d}^{\eta }Pu\;\text{d}Y+\int_{-d}^{\eta }Eu\;\text{d}Y=\int_{-d}^{0}Pu\;\text{d}Y+O(a^{3}). \end{array}$$

The average energy propagation $E_{f}={\bar{E}}_{f}$ can be evaluated to get

$$E_{f}=\frac{g^{2}a^{2}k}{4\omega \cosh^{2}(kd)}\left[d+\frac{\sinh(2kd)}{2k}\right]=\frac{1}{2}ga^{2}\cdot \frac{g}{2\omega }\left[\tanh(kd)+\frac{kd}{\cosh^{2}(kd)}\right]\overset{\left(\text{A}\;4\right)}{=}E\cdot c_{g}.$$

Hence, the mean-energy flux for a linear water wave is given by $E_{f}=E\cdot c_{g}$: the mean-energy density is propagated with the group velocity $c_{g}$.
